# P13 Understanding MDR *Escherichia coli* UTIs in the elderly: strategies for antimicrobial stewardship—from laboratory evidence to clinical practice

**DOI:** 10.1093/jacamr/dlaf230.020

**Published:** 2025-12-04

**Authors:** Aser Waleed Mohamed Marzok, Rasha Abdelsalam Elshenawy

**Affiliations:** School of Health, Medicine and Life Sciences, University of Hertfordshire, Hatfield, UK; School of Health, Medicine and Life Sciences, University of Hertfordshire, Hatfield, UK

## Abstract

**Background:**

Urinary tract infections (UTIs) caused by MDR *Escherichia coli* present a growing challenge in elderly patients, often leading to limited therapeutic options and poor clinical outcomes.^1^ Laboratory studies of *E. coli* resistance patterns can guide rational prescribing, but clinical decision-making must also account for patient-specific factors, comorbidities and antimicrobial stewardship (AMS) principles.^2,3^ This study bridges laboratory findings on *E. coli* resistance with real-world clinical application in an elderly patient with MDR *E. coli* UTI.

**Objectives:**

To evaluate antibiotic susceptibility trends in *E. coli* using the agar plate method under controlled laboratory conditions and to apply these findings to a real-world clinical case of a 72-year-old female with MDR *E. coli* UTI, highlighting AMS-guided therapeutic decision-making.

**Methods:**

The laboratory phase involved agar plate diffusion assays testing broad- and narrow-spectrum antibiotics (ampicillin, chloramphenicol, cefoxitin, erythromycin) against *E. coli*, with inhibition zones measured over four weeks to monitor susceptibility trends. In the clinical phase, we reviewed microbiology results from a 72-year-old female with symptomatic UTI, whose urine culture revealed *E. coli* (>100 000 cfu/mL) resistant to multiple β-lactams, fluoroquinolones and third generation cephalosporins, but sensitive to sulfonamides, carbapenems, nitrofurantoin and tetracyclines.

**Results:**

Laboratory findings demonstrated variability in *E. coli* susceptibility, with ampicillin showing reduced efficacy over time, cefoxitin maintaining stable activity and erythromycin demonstrating no activity. Clinical findings showed resistance to ampicillin, ciprofloxacin, levofloxacin, amoxicillin/clavulanate, piperacillin/tazobactam and most cephalosporins, with susceptibility retained to trimethoprim/sulfamethoxazole, imipenem, meropenem, nitrofurantoin, tetracycline, doxycycline and ertapenem. These results suggest a MDR strain likely producing ESBLs (Figure 1).

**Conclusions:**

Integrating laboratory resistance surveillance with clinical case evaluation supports targeted antibiotic therapy in MDR *E. coli* UTIs, particularly in vulnerable elderly populations. For the presented patient, oral nitrofurantoin or trimethoprim/sulfamethoxazole could be appropriate first-line agents for lower UTI, with carbapenems reserved for complicated or systemic infections. Where a broad-spectrum agent is initiated empirically, timely de-escalation to the narrowest effective oral option based on culture results is essential to reduce selective pressure and minimize collateral damage to the microbiome. This combined approach emphasizes the importance of continuous local susceptibility monitoring, prompt review of microbiology results, prudent antimicrobial selection and adherence to AMS strategies (Figure 2)—including early de-escalation, shortest effective treatment duration and avoidance of unnecessary broad-spectrum use—to optimize patient outcomes, prevent recurrence and slow the progression of antimicrobial resistance.

**Figure 1.**
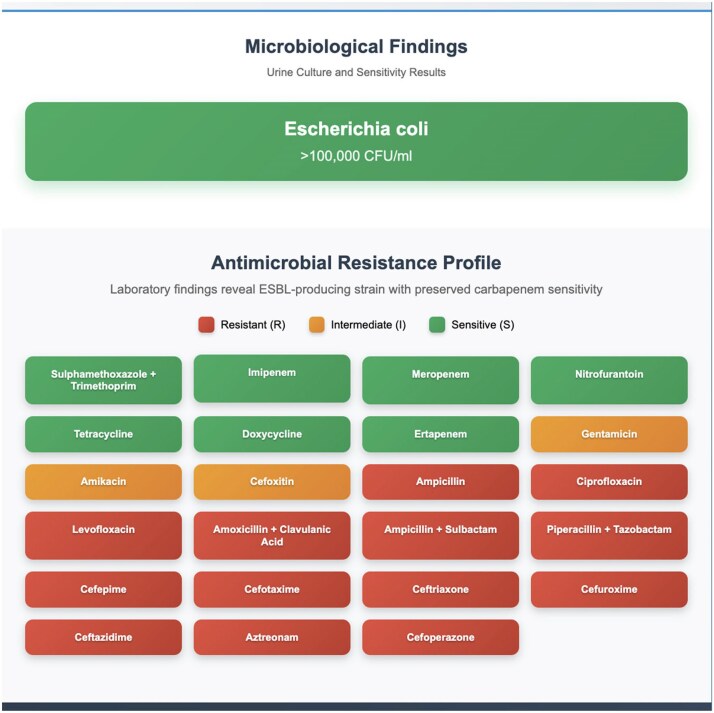
Antimicrobial resistance profile of *Escherichia coli* isolate from a 72-year-old patient, showing ESBL production and preserved carbapenem susceptibility.

**Figure 2.**
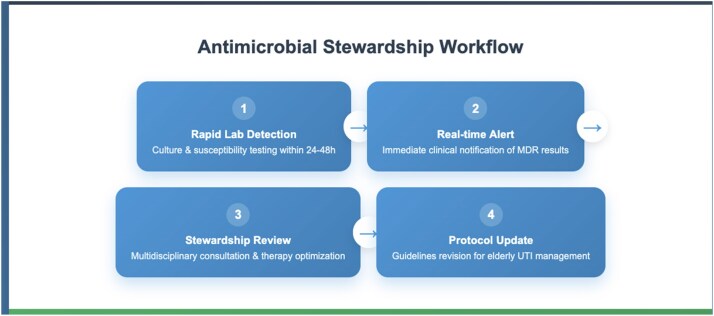
Proposed antimicrobial stewardship workflow for the rapid detection, review and protocol optimization in elderly UTI management.
